# To fall or not to fall: the question of hip fracture prevention

**DOI:** 10.1093/jbmr/zjaf198

**Published:** 2025-12-18

**Authors:** Karl J Jepsen

**Affiliations:** Department of Orthopaedic Surgery, School of Medicine, University of Michigan, Ann Arbor, MI 48109, United States; Department of Biomedical Engineering, College of Engineering, University of Michigan, Ann Arbor, MI 48109, United States

**Keywords:** bone, fracture, osteoporosis, falls, hip

To fall or not to fall, that is the question addressed by Johannesdottir and colleagues^1^ in their paper titled “The relationship between fall risk, trochanteric soft tissue thickness and hip fracture risk in older adults.” In this study, the authors developed a fall risk tool and showed that including the likelihood of future falls improved hip fracture risk prediction beyond that provided by the FRAX score alone.

Falls are the leading cause of injury-related deaths among community-dwelling individuals older than 65 years of age.^2^ The importance of falls to hip fractures is well recognized.^3^ Many individuals live in fear of falling because an incident hip fracture is associated with a high risk of mortality or changes in quality of life, mobility, and independence. Although more than 98% of hip fractures arise from a fall, only 1%-2% of falls lead to a fracture,^4^^,^^5^ because the direction of falling affects the likelihood a fall results in a fracture.^5^ A history of falling is associated with increased fracture risk independent of BMD and with effect sizes that are greater in men than women, unaffected by race and ethnicity,^6^ and decline with age.^7^ Johannesdottir and colleagues analyzed the association between falls and hip fractures in a new way by including the probability of future falls into their fracture risk model. Because falling to the side increases the likelihood of converting a fall into a fracture,^5^ Johannesdottir and colleagues also included trochanteric soft tissue thickness (TST) in their models.

In the first analysis, baseline data on older women and men enrolled in the longitudinal Age, Gene/Environment Susceptibility (AGES) Reykjavik Study who completed fall questionnaires at baseline and follow-up visits were used to predict repeated falls over 5 years. A “faller” was defined as someone reporting more than 2 falls in the prior 12 months. Factors that predicted future falls included age, being female, low scores in several physical functioning tests, use of medications with known associations with falls, and not surprisingly a history of falling. Of these, the timed-up-and-go (TUG) test was the strongest predictor of future falls along with fall history and grip strength. From these multivariable models, they developed subject-specific fall risk scores reflecting the probability of falling.

In the second analysis, they conducted a case-cohort study of randomly selected individuals from AGES and tested if combining the probability of falling and TST with FRAX with BMD predicted hip fractures better than FRAX with BMD alone. The probability of falling predicted hip fractures in women and men better than FRAX alone, with the effect being stronger in men than women. The worst 2 quintiles of the fall risk scores were associated with increases in hip fractures of ~2 fold for women and 4-8 fold for men independent of FRAX with BMD. Including the probability of falling with FRAX improved the AUC by 6%-8% in women and 13%-20% in men over 16 years. Not surprisingly, having lower TST was associated with increased hip fracture risk, an effect that was significant in women but not men.

To summarize, two individuals can have similar BMD values but the one who will fall more will have a higher likelihood of sustaining a fracture. This outcome may seem obvious, but there are important clinical implications to note. Clinical diagnostics rely on measures like BMD to assess bone health and fracture-mitigation interventions are often targeted to improve BMD and by extension bone strength. Despite age-related bone loss, our skeletons are, in general, sufficiently strong and well adapted to support loads associated with daily activities. However, a sideways fall loads the hip in a direction that is not part of daily activities. Consequently, the structure of the aging proximal femur is not well adapted to resist fracturing under these aberrant loads. Johannesdottir and colleagues did not control for osteoporosis medication use, so it is unclear if the BMD of those in the fracture or control cohorts represented age-related bone loss. Nevertheless, the results may suggest that interventions aiming to slow further bone loss or rebuild lost bone may only go so far with reducing fractures and that intervention options should also be more intentional about improving balance and mobility.^8^ By showing the probability of falling was associated with 2- to 13-fold increase in fracture risk independent of FRAX with BMD, one could argue that clinical diagnostics should also include physical functioning measures like the TUG test which assesses a person’s mobility, balance, and walking ability.^9^

Focusing on predictors of future falls highlights the need to expand the development of early indicators of fracture risk. Currently, most clinical diagnostics are reactive (ie, lagging indicators) in that they can detect a bone health problem once there is loss of mass (aBMD), loss of strength, a fall has occurred, or a fracture has occurred. The fall risk score moves the field one step closer toward providing proactive personalized diagnostics (ie, leading indicators) that also provide for informed decision making on intervention options. The ability of fall history to predict fracture risk diminishes with aging in women^7^ and so it will be important to develop fall risk models that can be updated since a crystal ball can only see so far into the future. From this author’s perspective, anything that can be done to reduce future falls and improve confidence and quality of living would be an impactful investment in fracture prevention strategies. The timed-up-and-go tests can be conducted at home to monitor the effectiveness of exercise regimens for reducing the likelihood of a fall. Anecdotally, people just want to know how to make best use of their time devoted to exercise and to know that what they do will pay off later in life with lower fracture risk. If “all the world’s a stage,” then further development of the fall risk score to include multiple races, ethnicities, and age-groups may expand the various roles of bone building and fall reducing interventions and their effectiveness in lowering hip fracture incidence.
